# Genetically Modified Corn— Environmental Benefits and Risks

**DOI:** 10.1371/journal.pbio.0000008

**Published:** 2003-10-13

**Authors:** Virginia Gewin

## Abstract

To plant or not to plant. A discussion of the environmental benefits and risks of genetically modified crops

Corn is one of humankind's earliest innovations. It was domesticated 10,000 years ago when humans learned to cross-pollinate plants and slowly turned a scraggly nondescript grass called teosinte into plump, productive modern corn (Figure 1). As needs change, so does plant breeding. Today, while biotech super-giants manipulate corn genetics to satisfy farmer desires and a global market, indigenous Mexican farmers do so to fulfill individual needs. Although the tools differ, the goal remains the same—to cultivate desirable traits.

**Figure 1:**
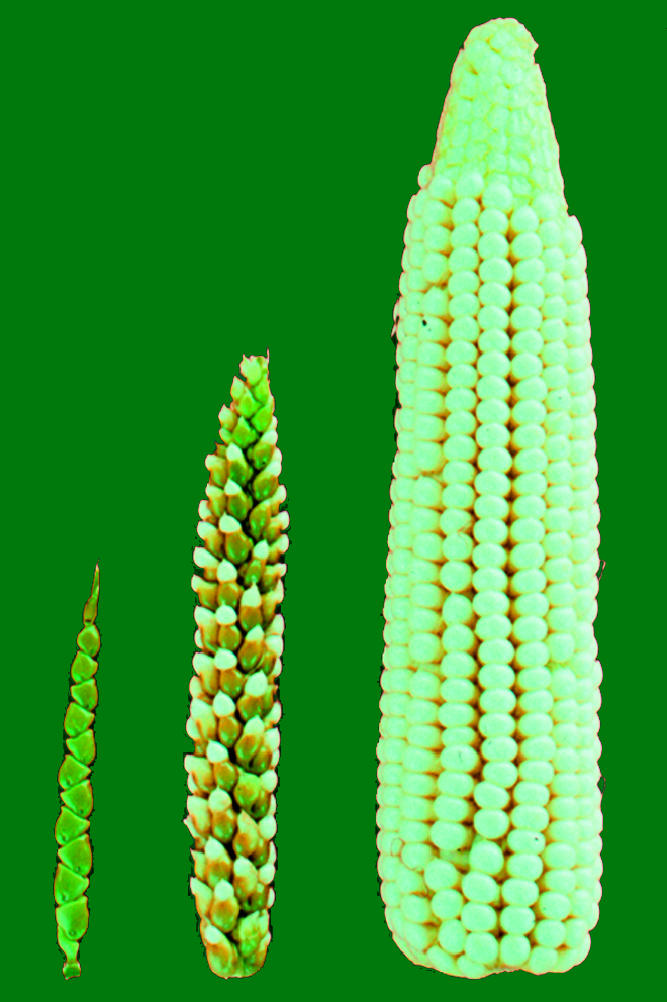
Crossing for Kernels Over time, selective breeding modifies teosinte's few fruitcases (left) into modern corn's rows of exposed kernels (right). (Photo courtesy of John Doebley.).

Plant breeding was once restricted to sexually compatible plants, and generations of offspring were selectively bred to create unique varieties. In fact, corn, along with rice and wheat—today's global crop staples—would not exist without such techniques. With the goal of ever-widening the pool of genetic diversity, conventional plant breeding has gotten more technologically savvy in recent years. For example, realizing that natural mutants often introduce valuable traits, scientists turned to chemicals and irradiation to speed the creation of mutants. From test-tube plants derived from sexually incompatible crosses to the use of molecular genetic markers to identify interesting hereditary traits, the divide between engineering and genetics was narrowing long before kingdom boundaries were crossed.

But when geneticists began to explore microorganisms for traits of interest—such as Bacillus thuringiensis (Bt) genes that produce a protein lethal to some crop pests—they triggered an uproar over ethical, scientific, and environmental concerns that continues today. (See Box 1.)

**Box 1. Bt Technology**

Bacillus thuringiensis, a soil bacterium, produces several crystal (Cry) protein toxins that destroy the gut of invading pests, such as larval caterpillars. So far, over 50 *cry* genes have been identified and found to affect insect orders differently.

Considered safe to humans, mammals, and most insects, Bt has been a popular pesticidal spray since the 1960s because it had little chance of unintended effects. Engineering the gene into corn, however, caused an unexpected public backlash. “We thought it was going to be the greatest thing since sliced bread,” says Guy Cardineau, agricultural biotechnologist at Arizona State University. “Here's a way to withstand insect pressure, eliminate the use of pesticides, and Bt spray was widely used in organic agriculture,” he adds. The Bt wrangle illustrates how differently a product and a process can be regarded.

After the expensive development process, today's concern is that broad-scale planting of Bt corn will render the toxin ineffective over time. Pests can gradually build resistance to any pesticide, and so the United States Environmental Protection Agency (EPA) requires that 20% of Bt field areas be planted to non-Bt corn to avoid such pressures. But humans have to follow the rules. A recent report from the Center for Science in the Public Interest shows that almost 20% of farmers in the United States Corn Belt are violating EPA standards by overplanting Bt corn, causing some to question the regulations and enforcement that will be necessary for certain GM crops.

Despite such discord, genetically modified (GM) crops have the fastest adoption rate of any new technology in global agriculture simply because farmers benefit directly from higher yields and lowered production costs. (See Table 1.) To date, the two most prevalent GM crops traits are Btderived insect resistance and herbicide resistance.

**Table 1:**
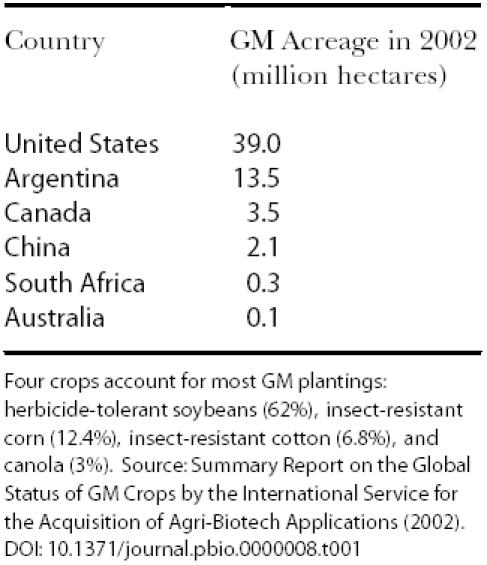
Worldwide production of GM crops

Four crops account for most GM plantings: herbicide-tolerant soybeans (62%), insect-resistant corn (12.4%), insect-resistant cotton (6.8%), and canola (3%). Source: Summary Report on the Global Status of GM Crops by the International Service for the Acquisition of Agri-Biotech Applications (2002)

Since 1987, over 9,000 United States Animal and Plant Health Inspection Service (APHIS) permits have been issued to field-test GM crops. According to APHIS, corn is the most tested plant. The International Service for the Acquisition of Agri-Biotech Applications confirms that biotech corn is the second-most common GM crop (after soybean), with 12.4 million hectares planted in 2002. GM corn starch and soybean lecithin are just two of the ingredients already found in 70% of the processed food supply.

With future incarnations on the horizon, GM corn remains a lightening rod for debate. Embroiled in numerous controversies, corn has become biotech's boon and bane.

## Benefits Emerging

As Danforth Center President Roger Beachy, the first to develop a virus-resistant tomato, describes it, the first-generation GM crops were intended to help farmers reduce not only the impact of pests, but also the use of agrochemicals in modern crop production–a legacy of the Green Revolution. After a decade of cultivation, environmental benefits are emerging.

Bt corn reduces the need for pesticides, and while the primary benefit comes largely during a heavy corn-borer infestation, an unpredictable event, a secondary effect is that beneficial insects fare much better under these conditions. The numbers are particularly impressive for Bt cotton: the spraying of almost 2 million pounds of pesticides—roughly 50% of previous usage—has been spared since the large-scale adoption of Bt cotton.

According to Leonard Gianessi, senior research associate at the National Center for Food and Agricultural Policy, farmers who adopt GM crops make more money in tougher times. Indeed, insect- and virus-resistance traits have already saved several industries. Bt cotton is credited with reviving the Alabama cotton industry, hard hit by uncontrollable bollworm infestations. Likewise, genetically engineered papaya, made resistant to the papaya ringspot virus, salvaged Hawaii's fifth largest crop industry.

Herbicide-resistant crops engendered a different reception. While GM critics acknowledge that the use of a more benign herbicide, called by its trade name Roundup, can have environmental benefits, the creation of a market monopoly is a key criticism. However, the increased planting of herbicide-resistant soybeans is an integral, but not sole, factor in the increased adoption of no-till farming— a strategy that reduces soil erosion.

Surprise benefits have also occurred. According to the recent International Council for Science (ICSU) review of GM crops, disease-resistant corn crops may have lower levels of mycotoxins, potentially carcinogenic compounds to humans. They result from fungal activity in insect-infested corn crops. With fewer insect holes in plant tissue, associated fungi are not able to invade and produce toxins.

While there is a growing amount of data documenting the intended environmental benefits of GM crops, the potential risks are more elusive.

## Risky Business

After seven years of GM crop production and no apparent health effects, potential environmental risks—particularly gene flow into other species—have eclipsed food safety as a primary concern. As pollen and seeds move in the environment, they can transmit genetic traits to nearby crops or wild relatives. Many self-pollinating crops, such as wheat, barley, and potatoes, have a low frequency of gene flow, but the more promiscuous, such as sugar beets and corn, merit greater concern.

Determining where genes flow is a thriving research avenue, but the real question becomes “so what?” The risks associated with gene flow—such as creating weeds from introduced traits, reducing biodiversity, or harming nontarget species—are similar to those from conventionally bred crops. “I wouldn't dismiss the ecological concerns out of hand, but I think they can be exaggerated,” says Gabrielle Persley, the ICSU report author.

There are few instances of crop plants becoming weeds. Bred so intensely for hundreds of years, most crops cannot survive without human intervention. Increased weediness could be conveyed, however, if the plants are more fit or able to out-compete other crop species by producing more seed, by dispersing pollen or seed further, or by growing more vigorously in a specific environment. In fact, transgenic sunflowers can produce over 50% more seed than traditional varieties. Additionally, recent work shows that, compared to pollen, seeds are more likely to spread GM sugar beet genes into wild relatives in western Europe. Norman Ellstrand, plant geneticist at the University of California at Riverside, has shown that gene flow from many conventionally bred crops increases the weediness of nearby wild relatives.

For many domesticated crops, wild varieties do not exist in current areas of cultivation. Nevertheless, regions where crop species originated are particularly vulnerable to transgenic gene flow into local varieties, or landraces. Some fear that transgenic varieties with a competitive advantage might gradually displace valuable genetic diversity. For these reasons, transgenic corn is prohibited in Mexico, home to over 100 unique varieties.

Despite the ban, transgenes have been found in Mexican corn. “We have in several instances confirmed that there are transgenes in landraces of maize in Oaxaca,” says Ariel Alvarez-Morales, plant geneticist at the Mexican Center for Research and Advanced Studies (CINVESTAV) in Irapuato. The ramifications of this will not be known for some time, but Luis Herrera-Estrella, CINVESTAV's Director of Plant Biotechnology, is convinced that these single gene traits will be of little consequence to native Mexican varieties. “If Bt genes give an advantage to the farmer, they will keep growing it. In that case it will not be bad,” he says of dynamically changing landraces. “Gene flow has been occurring for 50 years from commercial lines to landraces.” While admitting this, Ellstrand points out that “if there are genes that you don't want to get into landraces—this shows how easily they can get there.” (See Box 2.)

**Box 2. Pharma Corn**

“The gene flow risk that keeps me awake at night is the possibility of hybridization between crops engineered to manufacture poisons and related crops intended for human consumption,” says plant geneticist Norman Ellstrand. Indeed, this application of GM crops seeks to turn corn into cost-effective pharmaceutical factories and may bear the mark of unacceptable risk. It is currently the subject of intense debate. An open-pollinated crop, corn is known for its promiscuity—making it more prone to gene flow risks than other crops. Genetic contamination takes on a whole new meaning when the escapable trait could produce proteins to treat diabetes or a hepatitis B vaccine.

Given that pharma corn demands multiple safety measures—including production in remote areas, separate farm equipment, delayed planting to offset pollination—many ask, “Why use corn?” “We know so much about corn genetics,” explains agricultural biotechnologist Guy Cardineau, “and it naturally lends itself to production with kernel packets of protein that can be stored indefinitely.” A number of scientists and United States food makers are not yet convinced that the benefits outweigh the risks and have joined environmental groups in questioning the use of pharma corn.

Over 130 acres of pharma crop field-tests were planted in 2002. Several products have moved on to clinical trials. Aware of concerns, the members of the influential Biotechnology Industry Organization decided last December to overturn its initial decision to remove pharma crops from the United States Corn Belt states and voluntarily police their use. A Colorado trial of corn producing a protein to treat cystic fibrosis recently began.

Indeed, unintended impacts are a primary concern. The potential risk to nontarget organisms took center stage when a 1999 paper in *Nature* suggested monarch butterfly populations might be adversely affected by Bt transgenes. Corrected by subsequent publications, the field experiments did not support original laboratory results. But effects on other nontarget organisms, such as soil microbes, remain a concern. When microbial genetics research uncovered how genes could be transferred between species in ways other than reproduction, so-called horizontal gene transfer, it not only explained why microorganisms were so diverse, but that microbes could potentially be endowed with GM plant DNA found in the soil. “Although a theoretical possibility, there is no evidence that it happens at any degree of frequency to be meaningful,” says Persley.

Opinions differ on this, however, and seem to follow the United States–European Union divide over the use of GM crops. Kaare Nielsen, microbial geneticist at Norway's University of Tromsø, is one of the few scientists to find examples of horizontal gene transfer. “There are actually very few studies and most of the ones conducted have been on first-generation plants,” Nielsen explains. Given that plant DNA can last in soil for over two years, Nielsen does not believe the possibility can be dismissed and argues that long-term studies are necessary. Work continues in this area in Europe.

The lack of baseline ecological data—even agreeing on what an appropriate baseline is—presents a substantial knowledge gap to environmental impact assessments. Scientists, including Nielsen, wonder whether there could be unexpected risk factors. Allison Snow, weed expert at Ohio State University, agrees with what many feel is the most important risk—the inability to anticipate all the effects. “Do we know all of the right questions we should be asking?” she wonders, adding, “Genes are complicated and can interact.” For these reasons, identifying factors that regulate weed and pest populations and determining how microbial community changes affect larger ecosystems are important areas of research.

## Do Risks Differ for Developing Nations?

To two academicians that kindled the biotech revolution, the real GM risks lie in how science is misinterpreted and misused. In fact, much of the currently conducted basic research is not likely to be applied in the near future. Public concerns coupled with corporate consolidation created huge roadblocks, especially in getting the technology to developing nations. While Beachy blames the skyrocketing regulatory costs that “are due to regulators who have not put into context this technology and its relative safety,” Richard Jefferson, chairman and chief executive officer of the Center for the Application of Molecular Biology to International Agriculture in Australia, fears that innovation has been stifled by corporate short-sightedness. “The biggest risk is that [biotechnology] maintains itself as a monolithic, expensive industry with untenable entry barriers for smaller enterprises,” he says.

Indeed, when does the risk of not using available technology factor into the debate? (See Box 3.) Many scientists argue that genetic modification can help to ensure food security in developing countries, specifically in Africa. While more than 25% of the 2002 global biotech acreage was grown in countries such as Argentina, China, and India, there is little applied research on crops relevant to famine-prone African countries.

**Box 3. Golden Rice**

Current regulatory constraints have a choke-hold on innovations for genetic modifications that seek to improve subsistence crops, such as rice. Golden rice, yellowed in appearance because it is infused with the vitamin A precursor beta-carotene, could save thousands of malnourished people each year from blindness and the other vitamin A–deficiency diseases prevalent in Southeast Asia.

Intellectual property issues and opposition from anti-GM activists have confounded the development for years. Faced with patent issues and regulatory hurdles and costs, developer and academic researcher Ingo Potrykus formed an alliance with Syngenta (then AstraZeneca Corporation) to allow the free licensing of the patents to public research institutions for humanitarian use. In addition, farmers making less than US$10,000 will receive free golden rice seed.

After over a decade of work, golden rice is still not on the market. The retired Potrykus is determined to bring this technology to farmers once it passes regulatory field testing—an additional four-year delay that he feels is scientifically unnecessary. “Nobody can construct even a hypothetical risk to the environment from golden rice,” he says, adding that nutritional risks are nonexistent as well. He acknowledges, however, that field tests will be beneficial for acceptance of this and future bio-fortified products. “I have experienced so much support in these countries, I don't think it [the anti-GM lobby] will be able to stop this project once it passes regulation,” he says.

“Food security is not going to come from crops being marketed outside Africa, like wheat or rice,” says John Kilama, Uganda native and president of the Global Bioscience Development Institute. He points out that of traditional staple crops such as cow peas and millet, only cassava has merited some publicly-funded research. Beachy estimates that it takes between US$5 million and US$10 million to bring a GM crop to market. Given regulatory costs, neither industry nor universities can afford to develop products that do not have mass appeal. “If the crop is not worth that much to the seed market, it's likely that we'll never see the varieties because of the cost of regulation,” he says.

To ensure a return on research investments, with the regulatory costs often the biggest ticket item, developing blockbuster traits is a priority. “Given the diversity of environments and cropping systems, there are not many more blockbusters such as Roundup resistance in the wings,” says Jefferson. The alternative, he adds, is to make it cheaper to innovate local varieties in ways that are likely to gain public acceptance. (See Box 4.)

**Box 4. Apomixis**

One way to minimize the problems associated with gene flow is to introduce sterility, such that pollen cannot transmit information. Richard Jefferson has high hopes for an accessible, cheap way for farmers to produce genetically superior seeds, called apomixis.

But similar concepts have been floated before. The controversial terminator technology prevented gene flow, but it also outraged activists because it kept farmers from reusing seed.

Unlike terminator, apomixis is “germinator” technology—avoiding fertilization altogether by producing seeds without pollination. In effect, seeds can be natural clones of the mother, instead of a genetic exchange between mother and father. Therefore, hybrid quality can be maintained as farmers use seed year after year.

Although apomixis occurs naturally in about 400 plant species, Jefferson believes that it can be successfully developed as a useful trait in other crop plants. To ensure its widespread availability, Jefferson and collaborators pledged not to create restrictive patent rights that could block the development of apomixis.

“The Green Revolution largely bypassed Africa,” says Josette Lewis, biotechnology advisor for the United States Agency for International Development. Given monetary constraints that prevent access to many biotechnologies, many scientists worry that the Gene Revolution might as well. Looming trade issues coupled with food insecurity shape the debate in Africa. Caught between the United States and European Union trade disputes, sub-Saharan countries are eager to use any technology that will enhance production without jeopardizing trade.

**Figure 2:**
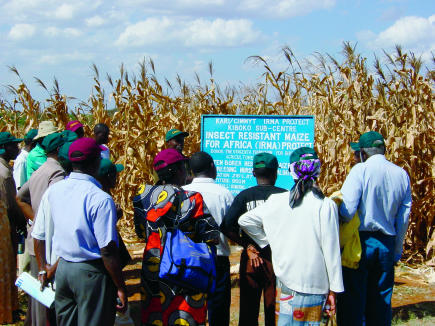
Biotech Bridge to Africa In an effort to reduce corn stem-borer infestations, corporate and public researchers partner to develop local Bt corn varieties suitable for Kenya. (Photo courtesy of Dave Hoisington/CIMMYT.).

Increasingly, industry is responding to the developing nations' needs. Both newly formed, the industry-focused African Agricultural Technology Foundation and the Public-Sector Intellectual Property Resource for Agriculture are attempting to ease cost restrictions and encourage access to current and future patents. By entering into such agreements, industries will be able to protect patent rights and commercially important markets. Such partnerships are already working. The Syngenta Foundation for Sustainable Agriculture is working together with the International Maize and Wheat Improvement Center (CIMMYT) and the Kenyan Agricultural Research Institute to overcome corn stemborer infestations in Kenya (Figure 2). “CIMMYT hopes to have a handful of local Bt corn varieties in farmers' fields by 2008,” says the admittedly ambitious Dave Hoisington, director of CIMMYT's Applied Biotechnology Center. Collaborations between public and private sectors may be the only way to navigate thorny patent issues and research crop varieties of interest to developing countries.

## Conclusion

“Agricultural biotechnology is here to stay” read a recent opinion piece by Gianessi. No doubt he is correct. As genetic engineering continues to evolve, transgenic methods will become just one of many tools. In fact, some researchers are currently focusing their work on manipulating an organism's own genetic code to achieve desired traits.

Scientific inquiry will continue to weigh the risks and benefits of such technologies, realizing that there may never be enough evidence to ensure zero risk. Only with data will tolerable levels of environmental risks be determined—case by case.

Indeed, the level of risks and benefits may differ for developing nations, where decisions must be made in the face of food security concerns. To many scientists, the risks associated with forgoing genetic engineering far surpass any environmental risk associated with its use and further development. However, all stakeholders must have access to the tools in order to realize future benefits.

In the quest to feed the world, a few things are clear. Public outcries will continue to vet the need and use of genetic engineering. Private organizations will necessarily focus on research for profit, while exploring collaborative prospects. Public initiatives, however, will provide the critical bridge between science and global food security.

Although genetic engineering cannot be summarily accepted or rejected, any lack of scientific risk now doesn't negate future concerns. And, no matter what direction future research takes, corn will continue to be a bellwether crop.

## Abbreviations

APHIS: United States Animal and Plant Health Inspection Service
Bt: Bacillus thuringiensis
CIMMYT: International Maize and Wheat Improvement Center
CINVESTAV: Center for Research and Advanced Studies
EPA: Environmental Protection Agency
GM: genetically modified
ICSU: International Council for Science.

